# Mechanisms of Maggot-Induced Wound Healing: What Do We Know, and Where Do We Go from Here?

**DOI:** 10.1155/2014/592419

**Published:** 2014-03-13

**Authors:** Ronald A. Sherman

**Affiliations:** BioTherapeutics, Education & Research (BTER) Foundation, 36 Urey Court, Irvine, CA 92617, USA

## Abstract

Medicinal maggots are believed to have three major mechanisms of action on wounds, brought about chemically and through physical contact: debridement (cleaning of debris), disinfection, and hastened wound healing. Until recently, most of the evidence for these claims was anecdotal; but the past 25 years have seen an increase in the use and study of maggot therapy. Controlled clinical studies are now available, along with laboratory investigations that examine the interaction of maggot and host on a cellular and molecular level. This review was undertaken to extract the salient data, make sense, where possible, of seemingly conflicting evidence, and reexamine our paradigm for maggot-induced wound healing. Clinical and laboratory data strongly support claims of effective and efficient debridement. Clinical evidence for hastened wound healing is meager, but laboratory studies and some small, replicated clinical studies strongly suggest that maggots do promote tissue growth and wound healing, though it is likely only during and shortly after the period when they are present on the wound. The best way to evaluate—and indeed realize—maggot-induced wound healing may be to use medicinal maggots as a “maintenance debridement” modality, applying them beyond the point of gross debridement.

## 1. Introduction

Maggot therapy (sometimes called larval therapy) is the application of live fly larvae to wounds in order to aid in wound debridement (cleaning), disinfection and/or healing. A maggot infestation on a living vertebrate host is called myiasis. When that infestation is limited to a wound, it is called wound myiasis. Maggot therapy is basically a therapeutic wound myiasis, controlled in ways that optimize efficacy and safety. We control the myiasis by carefully selecting the species and strain of fly (the species most commonly used is* Lucilia (Phaenicia) sericata*), disinfecting the larvae, using special dressings to maintain the larvae on the wound, and integrating quality control measures throughout the process.

The most noticeable change in maggot-treated wounds is debridement: the dead (necrotic or gangrenous), infected tissues and debris are removed from the wound, and the wound bed is left looking clean and healthy. But ever since maggot therapy became a common practice [1], careful observers also noted other effects on the wounds: microbial killing (disinfection) and hastened wound healing (growth stimulation).

Scientific evidence for all three actions has been slow in coming. The first controlled clinical trials were not begun until 1990 [2], and it was not until just 10 years ago that the U.S Food and Drug Administration (FDA) first granted marketing clearance to medicinal maggots (Medical Maggots; Monarch Labs, Irvine, CA) as a medical device [3]. The indications for that product were limited to debridement. Clinical evidence of maggot-induced disinfection and growth stimulation was not strong enough to convince regulators at that time. But today, numerous clinical and laboratory studies demonstrate antimicrobial and/or growth-promoting activity. Some clinical studies do not demonstrate these effects; instead, they leave us with doubts about the clinical significance of the wound healing activities that we see in most other clinical and laboratory studies.

Several comprehensive reviews have been published over the past decade [4–6], and readers interested in a more detailed or historical perspective would be advised to seek out these references. This review differs from those earlier works in that it was undertaken to examine the best clinical and basic science evidence that exists today, so as to formulate a course of future research that might answer some of the clinical questions that still remain.

## 2. Methods

A thorough literature search was conducted, first using the National Library of Medicine search tool (“PubMed”) and the Cochrane and Wiley Online Library databases, using the terms:

 [“maggot” or “larva” or “larval”] and [“therapy” or “wound”]

Then, the holdings of the BTER Foundation's Biotherapy library were searched for complete copies of these and any additional publications on maggot therapy. Articles not already in the library's holdings were requested through interlibrary loans or directly from the authors. Irrelevant publications (i.e., natural myiasis rather than maggot therapy), nonquantitative case reports (fewer than 5 cases per publication) and simple reviews or news stories were then excluded from this working collection, along with articles older than 20 years. This time frame was selected because the first controlled clinical trial of maggot therapy, published in a peer-reviewed journal, appeared 17 years ago. Three abstracts were published prior to that, but they report on data subsequently published in peer-reviewed journals within the time frame of our literature search, so the data was captured that way.

From this working collection of data, and in the context of a larger body of literature and expert opinion going back 90 years, a cohesive scheme about maggot therapy was synthesized. This made it possible to suggest clinical trial designs that might bring us substantially closer to understanding the clinical utility of maggot therapy.

## 3. Results and Discussion

Using the search terms of “maggot” (or “larva” or “larval”) and “therapy” or “wound,” a total of 8,303 publications were identified in PubMed, 644 in Wiley Online Library, and 8 in the Cochrane Library. After deleting duplicate and irrelevant articles and simple case reports or reviews, 97 articles met review requirements (Table 1).

The resulting body of literature provided both laboratory and clinical evidence to support all three actions associated with maggot therapy: debridement, disinfection, and growth stimulation. Nonsupportive data also was retrieved, though less commonly. The best way to consider the role of maggots in wound healing may be to first review the wound healing process in general and then to separately summarize the literature concerning each major wound healing effect of the maggots.

### 3.1. Wound Healing and the Chronic Wound

Wound healing is classically described as 4 distinct but overlapping physiological phases of repair and rebuilding: (1) homeostasis; (2) inflammation; (3) proliferation; and (4) remodeling and maturing [7]. With each phase, new cells are recruited into the area to perform the work, or cells already present alter their activity to secrete new cytokines or perform new duties, in response to changing conditions in the wound (bleeding, hypoxia, alterations in cytokine concentrations, etc.). When no longer needed, the cells undergo apoptosis and are removed or engulfed by other cells (i.e., macrophages). Normally, these four waves in the healing process progress quickly and smoothly, one into the next. But occasionally healing may stagnate, and the wound is said to be chronic. Wound healing may be trapped at any phase (or even while undergoing a combination of phases), but typically it is within the inflammatory phase: dead, infected debris may not be adequately removed from the wound bed, and/or it might not be possible for the body to eradicate the local infection, and/or the proteases and other destructive products of inflammation by clearing the newly formed cellular and extracellular matrix as fast as it is being laid down. It is in this context that debridement, disinfection, or cellular proliferation and migration are so important, for they can push the stagnant wound into the next phase of healing.

### 3.2. Debridement

Of the three described actions of maggot therapy, debridement (physical and chemical) is the best studied. Each maggot is capable of removing 25 mg of necrotic material from the wound within just 24 hrs [8].

**Figure 1:**
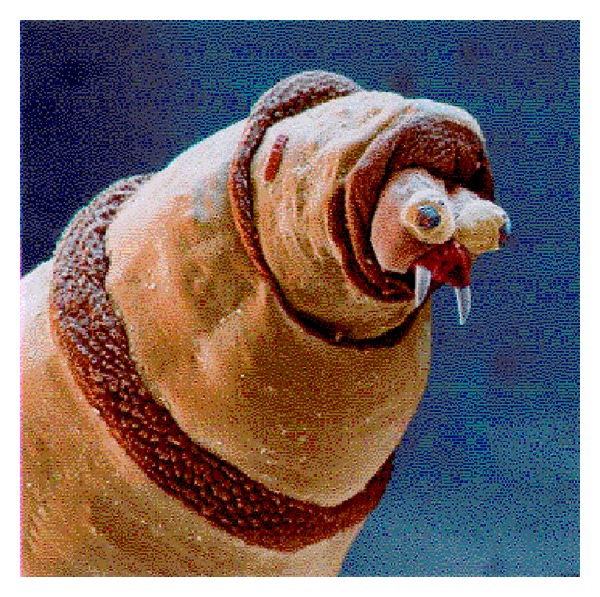
Scanning electron micrograph of* Lucilia (Phaenicia) sericata*. From Fleischmann W., Grassberger M., and Sherman RA Therapy. A Handbook of Maggot-Assisted Wound Healing. Stuttgart: Thieme, 2004:93 pg.

The physical mechanics of maggot debridement [6, 9] are readily apparent to anyone who has seen the larvae under the microscope. Larvae are covered by minute spines which scrape along the wound base as the maggots crawl about, loosening debris as does a surgeon's rasper or file (Figure 1). The mandibles, in the form of “mouth hooks,” are used to help pull the maggot's body forward as it crawls and to probe every nook and cranny for food or shelter. The maggot does not “bite off” pieces of tissue, but it rather secretes and excretes its digestive enzymes (alimentary secretions and excretions or ASE), the consequence of which is that digestion begins in the wound bed, outside of the maggot's own body. The necrotic tissue liquefies, and the maggots can then easily imbibe it. The physical movement of the maggot over the wound, plowing the tissue and spreading its ASE as it goes, contributes significantly to the debridement effort. The physical action of the maggot over the wound is a primary reason given by the FDA for classifying medicinal maggots as a medical device and not a simple drug.

Hobson [10] was one of the first investigators to systematically demonstrate proteolytic activity of* L. sericata* larval digestive enzymes. Vistnes et al. [11] used animal models to demonstrate that the maggots' digestive enzymes were capable of dissolving necrotic tissue and identified several proteases. More recent studies of larval ASE help us see just how these proteolytic enzymes fit into the context of debridement and wound healing, for we now know that they include a wide array of matrix metalloproteinases (MMPs), including at least the trypsin-like and chymotrypsin-like serine proteases, an aspartyl proteinase, and an exopeptidase-like MMP, active across a wide pH range [12–14].

It is important to recognize that humans produce at least 23 different MMPs which not only degrade extracellular protein but also regulate a wide variety of cellular processes through activation (or deactivation) of signaling molecules and/or their receptors [15]. MMPs play critical roles in all phases of tissue repair and wound healing, including hemostasis, thrombosis, inflammatory cell activation, collagen degradation, fibroblast and keratinocyte migration, and tissue remodeling. Disturbances in wound healing can occur when one group of proteases is deficient or out of balance with another.

Telford et al. [14] demonstrated that some of the maggot's proteases are resistant to human wound protease inhibitors. At least one of these chymotrypsin-like proteases has now been produced recombinantly in* Escherichia coli* [16] and could soon enter clinical trials as a purified debriding enzyme.

Larval secretions also contain deoxyribonuclease (DNAse), able to degrade both microbial DNA and also human DNA in necrotic debris [17]. DNAse may play an important role not only in debridement but also in inhibiting microbial growth and biofilm.

The wealth of case reports and case series in the literature suggests that most clinicians are impressed by the debridement efficacy of medicinal maggots. Controlled studies of maggot debridement are less common, but quite worthy of examination.

In a prospective study of spinal cord injury patients with chronic, nonhealing pressure ulcers, patients were monitored for 3-4 weeks while receiving standard wound care (whatever modality was prescribed by the surgically led wound care team), followed by 3-4 weeks of maggot therapy [2]. Tissue quality and wound size were assessed weekly. Maggot debridement of necrotic tissue was achieved in less than 14 days (average of 10 days), but none of the control wounds were more than 50% debrided, even after 4 weeks of treatment.

In a cohort of 63 patients with 92 pressure ulcers, followed for at least 8 weeks while receiving either standard wound care (as prescribed by the hospital's wound care team), or maggot therapy (two 48- to 72-hour cycles per week), maggot-treated wounds were debrided four times faster than control wounds (0.8 cm^2^/week versus 0.2 cm^2^/week; *P* = 0.001) [18].

In a similar cohort of 18 diabetic subjects with 20 nonhealing neuropathic and neuroischemic foot ulcers [19], maggot-treated wounds were 50% debrided within an average of 9 days, but control wounds did not achieve that level of debridement until an average of 29 days (*P* < 0.001). Within 2 weeks, maggot-treated wounds were left with only 7% necrotic tissue (0.9 cm^2^) compared to 39% necrotic tissue (3.1 cm^2^) in the control group (*P* < 0.01), and all maggot-treated wounds were completely debrided within 4 weeks, while most control wounds were still over 33% covered with necrotic tissue (*P* = 0.001).

Wayman and colleagues [20] randomized 12 venous stasis leg ulcer subjects to receive either maggot debridement therapy (MDT) or their standard of care (hydrogel). In this randomized controlled trial (RCT), the six wounds in the MDT arm were debrided faster than the six wounds in the control arm (*P* < 0.004), with all of the maggot-debrided wounds completely debrided after just one 2-3-day treatment, compared to only 4 of the control wounds completely debrided after a month of therapy.

In a larger clinical trial of maggot therapy for venous stasis ulcers, this time designed to look for maggot-associated wound healing, Dumville and colleagues [21] enrolled 263 subjects to receive either standard (“free-range”) maggot debridement, maggot debridement using “Biobags” (a patented ravioli-like pouch containing the live larvae), or their standard of care, hydrogel, and compression dressings (Figure 3). All subjects received compression dressings, except during maggot debridement. Time to debridement differed significantly between the three groups (25.38, df = 2, log-rank test *P* < 0.001). The median time to debridement was 14 days with free-range larvae, 28 days with bagged larvae, and 72 days for the control arm. Healing results will be discussed later in this review.

Most other debridement studies are not as quantitative in their data collection and assessments. Markevich and colleagues presented data from their RCT of maggot therapy for neuropathic foot wounds at the 2000 Conference of the European Association for the Study of Diabetes [22]. Although never published as a full-length, peer-reviewed research paper, this abstract is often cited because it is the only RCT of MDT in diabetic foot ulcers. Subjects were randomly assigned to receive maggot therapy (*N* = 70) or standard (hydrogel) therapy (*N* = 70). Wound dimensions and quality were then monitored every 3 days for 10 days. While the authors did not quantify debridement per se, we know that the maggot-treated patients were debrided more effectively and efficiently because their necrotic wounds were ultimately covered with more granulation tissue (*P* < 0.001) and were smaller in size (*P* < 0.05) than the wounds treated with hydrogel.

In a retrospective case controlled study of lower extremity wounds in nonambulatory hospice patients (in whom debridement was the goal, not wound healing) [23], Armstrong and colleagues concluded that MDT was an effective debridement modality. Again, their objective measures were not specifically changes in the amount of necrotic tissue but rather more clinically relevant surrogates: faster eradication of infection (127 versus 82 antibiotic-free days out of 6 months; *P* = 0.001), two-thirds fewer amputations (10% versus 33%; *P* = 0.03), and significantly faster wound healing in the maggot-treated wounds (18 weeks, for those that healed, versus 22 weeks; *P* = 0.04).

Marineau and colleagues [24] published their case series of 23 complicated diabetic foot wounds (most with osteomyelitis) treated with MDT. There was no control group and no analysis of individual wound changes, but the authors did conclude that the 74% of success rate (debridement or complete limb salvage) was greater than expected, given that this group of patients had all failed prior conventional wound care.

In their RCT of maggot therapy for chronic leg wounds, Opletalová and colleagues randomized 119 subjects to receive either surgical debridement or bagged maggots (twice weekly) for two weeks. Wounds were evaluated on days 8, 15, and 30 [25]. Wound slough was significantly less in the maggot-treated arm by day 8 (54.5% versus 66.5%; *P* = 0.04), but by day 15 that difference disappeared. The authors concluded that, compared to surgical debridement, maggot therapy was more efficient and valuable for the first 2 weeks, though additional treatments provided no debridement benefit.

This two-week limit to maggot debridement efficacy deserves comment and consideration, because it contrasts with what has been reported with free range maggots. Unfortunately, very few studies have compared free range with bagged maggots, though such a study could be a valuable mechanism for evaluating the relative importance of the maggot's physical versus chemical activity. Most, though not all, laboratory studies comparing free range versus contained maggots have suggested that maggots in direct contact with the wound are more effective, at least for debridement, than maggots separated from the wound by their containment dressings [9, 26]. To date, only one clinical study was designed to compare the difference between these two methods of maggot therapy. In this prospective clinical trial, Steenvoorde and colleagues [27] enrolled 64 patients with 69 chronic, necrotic wounds. Patients were treated with either free range or contained maggot debridement therapy, depending on maggot availability and clinician preference. The investigators monitored 8 specific outcome measures: (1) complete healing without any other intervention; (2) complete healing by secondary intervention (e.g., split-skin graft); (3) wound free from infection and less than one-third of the initial size; (4) wound clean but not decreased in size; (5) no difference in wound size or character; (6) wound worsened; (7) minor amputation was still required (e.g., partial toe amputation); and (8) major amputation was still required. Their analysis revealed better outcomes in the free range group compared to the contained maggots group (*P* = 0.028), despite the fact that the free range technique required fewer maggot applications (*P* = 0.028) and fewer total number of maggots per treatment (*P* < 0.001). The authors concluded that containment of maggots reduced the effectiveness and efficiency of maggot debridement therapy, probably by preventing contact with, and/or complete access to, the wound bed.

Dumville et al.'s study [21] discussed above included free range and contained maggots in two of the three study arms but was not specifically designed to detect differences in debridement between free range and contained maggots and did not identify any significant differences. The median time to debridement in this study was 14 days for the free range maggot therapy arm (95% confidence interval [CI] = 10–17) and 28 days for the bagged maggots (95% CI = 13 to 55; adjusted *χ* ^2^ 1.52, df = 1; *P* = 0.22). As pointed out, this study was not powered to detect significant differences between these two groups, so it is not possible to determine whether or not the twofold difference in debridement time is real.

### 3.3. Disinfection

The natural habitat of* L. sericata* larvae is in rotting organic matter such as a corpse or excrement. Therefore, it should be no surprise that this maggot would be well-protected from infection. Early on, scientists believed that ingestion was the primary method by which the maggots cleared the wounds of infection [8, 28], and subsequent researchers demonstrated that highly effective killing does indeed occur in the gut [29, 30]. Greenberg hypothesized that antimicrobial compounds might be produced in the gut by symbiotic microbes such as* Proteus mirabilis*, and, in 1986, Erdmann and Khalil identified and isolated two antibacterial substances (phenylacetic acid and phenylacetaldehyde) from the* P. mirabilis* that they isolated from the gut of a related blowfly larva:* Cochliomyia hominivorax* [31].

Antimicrobial killing also occurs outside the maggot's gut, and the extracorporeal secretion/excretion of antimicrobial compounds may even be responsible for most of the maggot's antimicrobial activity [32, 33]. Some early researchers believed that wound disinfection was largely due to the physical “washing-out” (lavage) of microbes from the wound bed during maggot therapy, by the fluid secreted by both the maggots (ASE) and the host (“wound exudate”). They also pointed to the antimicrobial activity of ammonia-containing byproducts of the maggots' digestion of tissue proteins and the resulting alkalinized wound bed [1, 34, 35].

With advanced molecular and biochemical methods now at our disposal, many researchers over the past two decades have focused their attention on isolating antimicrobial proteins and other biochemicals produced by* L. sericata* [36–47]. Often, the isolated molecules were more active against gram positive bacteria than gram negatives, but sometimes this was merely a matter of dose and potency [42]. Antimicrobial activity has been seen even against highly antibiotic-resistant bacteria [40, 43] and against the protozoan* Leishmania* parasite [44, 45]. Kawabata et al. [46] demonstrated that the antimicrobial activity could be modified by exposure to microbial challenges (as is the case with many innate immunodefense peptides).

By 2010, Cerovský et al. [47] completely sequenced the 40-residue defensin-like antimicrobial peptide now called: “lucifensin.” Altincicek and Vilcinskas [48] used suppression subtractive hybridization to show that 65* L. sericata* genes upregulated in response to septic challenge (cuticular puncture) with lipopolysaccharide. Valachová and colleagues [49] demonstrated that lucifensin expression was increased in response to microbial ingestion only in the fat body; lucifensin was expressed in the salivary glands throughout the larval period and not significantly affected by microbial ingestion.

Even more antimicrobial molecules are likely to be discovered in the coming years. Numerous antimicrobial molecules have already been isolated in other blow flies, including the antibacterial peptide diptericin from* Phormia terraenovae* [50] and the antiviral alloferons from* Calliphora vicina* [51], the latter of which has already been commercialized.

Maggots also fight bacteria in their more resistant form: biofilm. In contrast to free living (“planktonic”) individual bacteria, biofilm is a structured community of one or more species of bacterial cells, living closely in an enclosed, protective, self-produced polymeric matrix, and adherent to an inert or living surface [52]. Antibiofilm activity is valuable because biofilm is highly resistant to the penetration and successful activity of the human immune system and antibiotics. Biofilm is a particularly difficult problem in chronic wounds. One of the most powerful tools we have against biofilm is physically eroding it (i.e., brushing our teeth to rid ourselves of dental biofilm). Many therapists prescribe brushing to rid a wound of biofilm. It is reasonable to assume that the maggots are helping to rid a wound of biofilm simply by crawling over it with their rough bodies. What was particularly surprising, though, was the discovery that maggot ASE is capable of dissolving biofilm and inhibiting the growth of new biofilm [53–55]. This has been shown at least for* Staphylococcus aureus *and* Pseudomonas aeruginosa* biofilm.

There should be no more doubt that maggots secrete and excrete potent antimicrobial compounds. But what is the evidence that maggots bring about clinically relevant disinfection? Numerous case reports have purported wound disinfection following maggot therapy, but controlled clinical evidence of maggot-induced antimicrobial activity has been sparse, until recently. In a prospective clinical trial of maggot therapy for chronic leg ulcers, Contreras-Ruiz and colleagues [56] randomized 19 subjects to either maggot therapy or conventional debridement and compression therapy and found that maggot-treated wounds had significantly reduced bacterial counts compared to control wounds. The maggot-treated group displayed more anxiety and wound odor during treatment, but no greater pain or other adverse events.

In Tantawi et al.'s case series [57], 13 diabetic ulcers in 10 subjects similarly demonstrated significant decreases in the number of microbial species and the colony counts after maggot therapy. In an observational study by Bowling and colleagues [58], 13 sequentially enrolled stable diabetic patients with MRSA-colonized ulcers, not already receiving MRSA-specific antibiotics, were debrided with maggot therapy. Semiquantitative cultures were taken at baseline and before each cycle of MDT. The mean duration of MDT was less than 3 weeks (one treatment per week), and the authors noted that this was far less than the duration of conventional antibiotic treatment for MRSA. By the end of maggot debridement, MRSA colonization was eliminated from all but 1 of the 13 ulcers (efficacy = 92%); no complications or patient complaints were encountered.

When reviewing their patients, Steenvoorde and Jukema [59] also found decreased colony counts of gram positive organisms following maggot therapy, but they found increased counts of gram negatives. Their results may have resulted from the decreased competition by gram positive microbes. The study authors speculated that higher doses may be necessary for effective gram negative killing.

Armstrong et al. [23] probably best addressed the clinical relevancy of maggot-induced disinfection by designing a case-control study of maggot therapy for lower extremity wounds in hospice patients and recording the antibiotics prescribed by the patients' primary clinicians, as a measure of clinically significant infection. As described earlier in this review, this study revealed significantly fewer days of antibiotics compared to controls, over a 6-month observation period, indicating that the patients were cleared of their infection faster and remained infection free longer.

Not all clinical studies of maggot-induced disinfection have demonstrated such positive results. Dumville et al.'s 267-subject RCT of maggot therapy for venous stasis wounds [21] did not demonstrate any significant difference between the time-dependent decreasing bacterial burden in maggot-treated patients versus control patients, nor any significant difference in the number of MRSA-colonized wounds that were cleared. But then, as the authors pointed out, there were so few patients with MRSA that the study was not adequately powered to see any likely difference. What's more, looking for significant population differences in colonizing bacteria may not truly be an appropriate endpoint if we are really more concerned with clinical infections.

### 3.4. Growth Stimulation

Evidence of maggot-induced tissue growth or wound healing now comes from both laboratory and clinical studies and also suggests both mechanical and biochemical pathways. Among the early theories about maggot-induced wound healing were that the simple removal of debris and microbial killing [28] or the action of crawling over the clean wound bed [60] might be enough to stimulate wound healing. We now know that both of these hypotheses likely contribute to wound healing: physical and electrical stimulation of healthy cells can induce the release of host growth factors, and any meaningful reduction in debris and biofilm or microbial population likely decreases inflammation and promotes wound healing. Some investigators believed that the alkalinity of maggot-treated wounds, along with the isolated allantoin and urea-containing compounds, was responsible for wound healing [61]. In fact, today, allantoin and urea are components of many cosmetics.

With recent advances in cellular biology and chemistry, we now know that maggot ASE stimulates the proliferation of fibroblasts [62] and endothelial tissue (unpublished data), increases angiogenesis [63], and enhances fibroblast migration over model wound surfaces [64–66]. Biopsies of maggot-treated wounds reveal profound angiogenesis [67]. Using remittance spectroscopy to evaluate patients before and after maggot therapy, Wollina and colleagues [68] found that vascular perfusion and tissue oxygenation surrounding the wound actually increased following maggot therapy. Zhang and colleagues [69] are currently seeing evidence that maggot extracts may even stimulate the growth of neural tissue.

Early clinical reports of maggot-induced wound healing were merely case studies or series; but beginning in the 1990's, controlled comparative trials of maggot therapy began to appear. These were small, due to a lack of funding and support; but they showed the promising results needed to propel maggot therapy into the scientific limelight and justified larger and more definitive studies. In a prospective study of spinal cord injury patients with chronic, nonhealing pressure ulcers, patients were followed for 3-4 weeks while receiving standard wound care (whatever modality was prescribed by the surgically led wound care team), followed by 3-4 weeks of maggot therapy [2]. Tissue quality and wound size were assessed and photographed weekly. The average wound size (cm^2^) increased weekly during control therapy but decreased by over 20% per week with maggot therapy (*P* < 0.001). Debridement of necrotic tissue was achieved in just 10 days with maggot therapy. None of the control wounds were debrided by more than 50%, even with 4 weeks of treatment.

A cohort of 63 patients with 92 pressure ulcers was prospectively followed for at least 8 weeks while receiving either standard wound care (as prescribed by the hospital's wound care team) or maggot therapy (two 48- to 72-hour cycles per week) [18]. In patients with bilateral wounds, only one was treated with maggot therapy, and patients were allowed to select that one. Therefore, maggot-treated wounds tended to be larger (22 cm^2^ versus 14 cm^2^; P < 0.05) and deeper (35% down to bone in the maggot therapy group; 8% in the control group). Nevertheless, 4- and 8-week healing rates were significantly better for maggot-treated wounds than control wounds, as was the weekly decrease in surface area and the rate of granulation tissue growth over the base of the wound (see Table 2).

The wound healing rate, based on studies by Gilman [70] and Margolis et al. [71], was defined as the change in surface area divided by the mean circumference over time. Four and eight-week healing rates have repeatedly been shown to be accurate surrogates for wound healing in general, although they have not been accepted as substitutes for complete wound closure in clinical trials.

Indeed, twice as many wounds in the maggot-treated group completely healed during the period of observation (39% within an average of 12 weeks versus 21% within an average of 13.4 weeks). But most patients were not followed more than 10 weeks, and this difference was not statistically significant.

In another cohort of 18 diabetic subjects with 20 nonhealing neuropathic and neuroischemic foot ulcers, six wounds were treated with conventional therapy, six with maggot therapy, and eight with conventional therapy first and then maggot therapy [19]. As in the pressure ulcer patients, 4- and 8-week healing rates were significantly better for maggot-treated wounds than control wounds, as was the weekly change in surface area and the rate of granulation tissue growth over the base of the wound (Table 2). Repeated measures ANOVA indicated that treatment rendered was the only factor associated with these differences.

In Armstrong's retrospective case-control study of lower extremity wounds in nonambulatory hospice patients [23], in which the researchers demonstrated significantly better infection control and fewer amputations required in the maggot-treated group, the difference in wound healing rates between the maggot-treated group (57% healed) and the control group (33% healed) was not statistically significant. In this study population, the probability of healing may have had more do to with the patients' underlying circulatory compromise, malnutrition, and poor physiologic health than with the treatments rendered. For those wounds that did heal, wound healing was much faster in the maggot-treated wounds than in the control wounds (18 weeks versus 22 weeks; *P* = 0.04).

As previously discussed, in the 140-subject RCT by Markevich and colleagues [22], wounds treated with maggot therapy were ultimately covered with more granulation tissue (*P* < 0.001) and were smaller in size (*P* < 0.05) than the wounds in the control study arm. This 10-day long clinical trial failed to show any significant difference in wound healing between the MDT arm (60% healed by day 10) and the control arm (34% healed by day 10), but it is generally believed that the lack of any significant difference may be due to the fact that this 10-day debridement study was much too short to detect any meaningful wound healing. Indeed, 60% healing of diabetic foot ulcers in only 10 days instead of 10 weeks is, by itself, quite impressive.

Many in the wound care community looked with excitement at the study by Dumville et al. [21], intended to evaluate maggot-induced wound* healing* in venous stasis ulcers. This RCT demonstrated significantly faster debridement in the maggot therapy arms (as already discussed), but did not demonstrate any significantly faster healing in those subjects. Several reasons may explain this, including the simple fact that the maggots may not expedite healing in any clinically meaningful way. Alternatively, as the authors pointed out, their study may have been too small to demonstrate the difference, given that there were less than 100 subjects in each of 3 arms. Some believe that the reason that no greater wound healing was seen in the maggot-treated arms was related to the study design, which used a “maggot debridement” protocol rather than a “maggot growth promotion” protocol [72]. In this study, maggot therapy was stopped as soon as wounds were debrided (treatment day number 15, on average, for the free range maggot therapy group) and was never administered to those patients again, even if their wounds deteriorated over the subsequent 7 months that it took, on average, to heal [73].

Indeed, maggot-associated wound healing and antimicrobial activity is likely short-lived after the maggots are removed. Sherman and Shimoda [74] reported successful wound healing without infection or dehiscence in patients surgically closed 1–21 days following maggot debridement to be 100%, compared to wounds debrided without MDT or those debrided with MDT more than 21 days before closure, which healed successfully only 68% of the time.

Many clinicians intuitively feel that faster debridement brings faster wound healing. After all, the wound cannot heal if infected, necrotic tissue and debris are occupying the center of the wound. Yet, it has been difficult to find any large RCT that demonstrates this to be true [75]. Perhaps the problem has been that chronic wounds often reacquire infection or biofilm; and additional tissue may die, requiring redebridement. Addressing the on-going need for wound cleaning and disinfection is the paradigm behind “maintenance debridement,” and appears to be gaining support as an important strategy for treating wounds [76, 77].

If this paradigm is correct, it would explain why maggot therapy continued beyond the point of gross debridement has been associated with faster wound healing [2, 18, 19, 22]. It may be true that no one single method of maintenance debridement is faster than another. But maggot therapy is one of the few highly effective methods of debridement which can safely and inexpensively be continued throughout the healing process, which may explain why it remains one of the methods of maintenance debridement best associated with faster wound healing.

### 3.5. Miscellaneous Actions

Platelets, neutrophils, and monocytes/macrophages are among the first cells recruited to the young wound when they remain beyond their usefulness and contributed to an unending inflammatory phase that can interfere with or even prevent the wound from moving forward in the healing process. Maggot secretions have recently been found to affect the activity of these cells in ways that decrease inflammation. While this can be thought of as a subset of actions which promote wound healing, they are separated out for the purpose of this discussion because these actions may also play important roles in disinfection, if not also debridement.

Exposing unstimulated human neutrophils to crude* L. sericata* salivary gland extract, Pecivova and colleagues [78] measured no effect on superoxide generation or myeloperoxidase (MPO) release. But when opsonized zymosan stimulated neutrophils were exposed to high concentrations of the salivary gland extract, superoxide generation and MPO release were significantly reduced. The researchers concluded that medicinal maggots might aid in wound healing by decreasing the generation of proinflammatory factors in this way, while still maintaining normal phagocytosis or apoptosis.

van der Plas et al. [79] monitored cyclic AMP (cAMP) in human neutrophils before and after exposure to* L. sericata* ASE and then again in human monocytes [80]. Their findings of elevated cAMP and suppressed proinflammatory responses (without a measurable decrease in antimicrobial activity) led the authors to conclude that the larval secretions were moving the monocytes and neutrophils forward from the proinflammatory phase and into the angiogenic phase of wound healing [81].

Cazander and colleagues [82] recently discovered that maggot ASE reduced complement activation in healthy and immune-activated (postoperative) human sera by as much as 99.9% by breaking down C3 and C4 proteins.

### 3.6. Integrated Conceptualization of Maggot Therapy Actions

**Figure 2:**
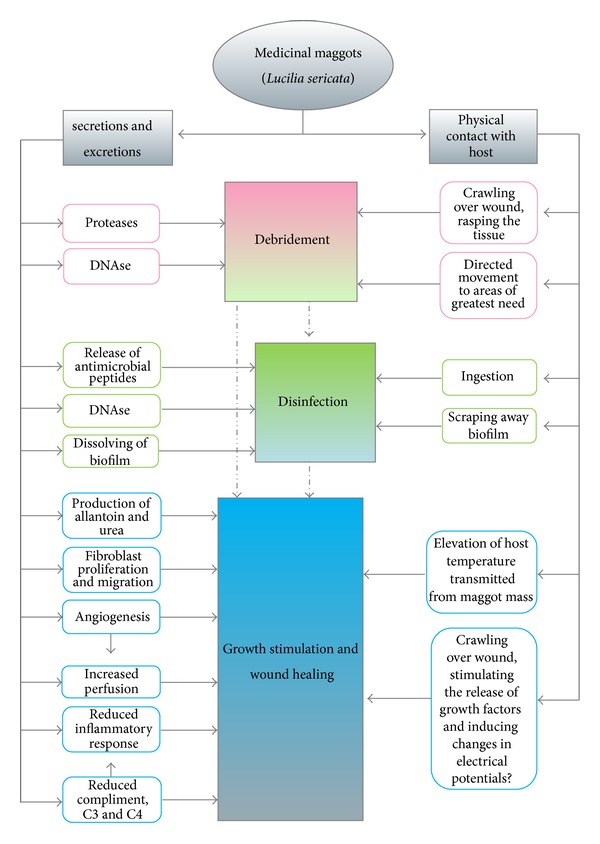
Schematic drawing of proven and postulated mechanisms by which medicinal maggots promote wound healing.

**Figure 3:**
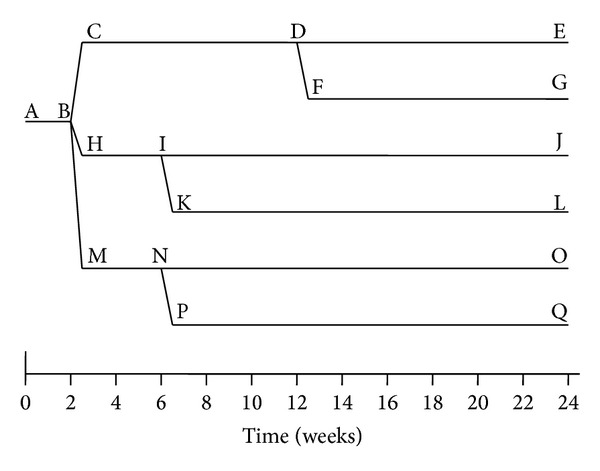
Schematic representation of a clinical trial proposed to demonstrate the wound healing effects of maggot therapy. After a 2-week baseline data collection (AB), nonhealing wounds are randomized either to receive the surgical and medical standard of care (CD), standard (confinement) maggot therapy dressings (HI), or containment (bagged) maggot dressings (MN) for debridement. Maggot-debrided wounds would then receive either standard care for wound closure (IJ; NO) or maggot therapy (MDT maintenance debridement, KL or PQ) to evaluate the presence of maggot-stimulated wound closure. To optimize enrollment and retention, subjects randomized to standard care may cross over to maggot therapy if there has been no significant improvement after 12–24 weeks of therapy.

From clinical and laboratory studies to date, it is clear that maggot therapy contributes significantly to wound care, both physically and biochemically.  Figure 2 represents our current understanding of the mechanisms by which maggot therapy affects wound healing. This schema is a work-in-progress, intended to be modified as additional research adds to our understanding of the maggot-wound interaction.

### 3.7. Future Study Recommendations

Many questions remain about wound healing, in general, and maggot therapy in particular. Several of these questions might be answered by a single well-designed clinical study. This review was undertaken to help design the next study or at least offer an initial proposal for what that study might look like.

Evidence of maggots' debridement efficacy is irrefutable. Clarity is still needed regarding maggot therapy's role in promoting wound closure. When maggot therapy has been used for debridement alone, some studies have shown faster overall healing, others have not. Those studies that have suggested or demonstrated significantly faster wound closure have looked at short-term findings: healing that occurs during or shortly after maggot therapy is administered. Studies that have looked at healing rates months after maggot debridement was terminated have not demonstrated any difference in healing rates. This is likely the key, for we now understand that maintenance debridement and maintenance disinfection can promote wound healing. We also now recognize that healthy-looking wounds can deteriorate quickly, especially when chronic, or when there are impediments to wound healing. The physical effects of maggots on the wound and the bioactive molecules that they secrete do not last long after therapy, so wounds that do not heal immediately after maggot debridement will be at risk for recolonization, infection, stagnation, and necrosis.

A single study might address these questions: Does maintenance debridement provide clinical benefits over single or episodic debridement, in terms of wound healing rates, and does maggot therapy enhance wound healing if administered as a maintenance debridement modality, that is, during and/or after complete debridement has already been achieved?

A randomized double cross-over study could address these questions if subjects were randomized to receive either maggot therapy or standard of care debridement, followed by standard of care or maggot therapy until wound closure. This 4-armed RCT would consist of the following: (1) standard of care throughout; (2) Standard of care debridement following by maggot therapy; (3) maggot debridement followed by standard of care thereafter; and (4) maggot debridement followed by maggot therapy maintenance (i.e., maggot therapy once weekly, ala Sherman et al., 2007 [83]).

The addition of two more study arms (or alternatively a separate study) could also address the advantages and disadvantages of free range versus contained (“bagged”) maggots. There is clear evidence that the long-touted benefits of maggot therapy are due, in part, to the physical contact of the maggots on the wound and the maggots' ability to mobilize to the deep recesses and other areas of need. A controlled comparative trial between free range and contained maggots would allow us to assess the relative contribution of the maggot's physical versus chemical contributions to wound healing.

Measurements of cost-efficacy, antimicrobial activity, and relative safety should also be incorporated into such a study, in order to capture as much data and address as many perspectives as possible concerning the clinical utility of maggot therapy for nonhealing wounds. This could be accomplished by collecting cost data for materials, services, and healthcare providers, collecting carefully selected and performed microbial cultures over the course of treatment, and monitoring a wide variety of health and quality-of-life parameters.

Such a large prospective study would be expensive and is not likely to be funded within the near future. Maggot therapy should not be withheld until such a study is completed, for there exists, already, a wealth of data supporting the efficacy and safety of maggot therapy in wound care. Smaller prospective studies and large registry studies may be able to address many of the same issues as does the RCT just proposed. But for those with the will and resources to conduct a large RCT of maggot therapy—even if such resources need to be pooled together—this is the RCT that might most efficiently yield the answers to the most pressing questions remaining about the mechanisms of maggot-induced wound healing.

## 4. Conclusions

Maggot therapy has long been recognized as a safe and effective treatment for wounds. It is associated with three broad actions: debridement, disinfection, and hastened tissue growth. We now know that these actions are the result of a large number of maggot-host interactions, some of them chemical and some physical. Essentially, the maggots crawl over the wound, plowing the base as they secrete their rich digestive enzymes, just as a farmer plows and fertilizes the field. Plowing without fertilizing or fertilizing without plowing, the farmer will produce a smaller yield and the maggots will be less effective in their debridement.

The maggots' secretions may even induce the maturation of monocytes and neutrophils from proinflammatory cells into their angiogenic phenotype, thereby lifting the wound out of its inflammatory rut, and then forward into the proliferative, healing phase of wound healing.

Today, the debridement efficacy and efficiency of medicinal maggots are beyond doubt. Debridement itself has been associated with both infection control and faster wound healing, yet the clinical utility of maggot-induced disinfection and growth stimulation activity remain suspect. Therapists can recount case after case of maggot-associated disinfection and wound healing, and most small clinical studies clearly demonstrate disinfection and/or growth stimulation alongside debridement. But the largest prospective clinical studies to date have demonstrated only maggot-induced debridement, not disinfection or growth promotion. Laboratory studies demonstrating disinfection and growth-promoting properties abound. Are we imagining a clinical effect that does not really exist? Or have we simply been unable to perform the RCT that would adequately and irrefutably demonstrate maggot-associated disinfection and wound healing?

Thorough review of the literature suggests that the debridement, antimicrobial, and growth-promoting activities may be short-lived, lasting no more than a few weeks after maggot therapy is terminated (not unlike the actions of most wound therapies). Debridement efficacy can be measured at the culmination of the maggot debridement treatment, but healing itself cannot be measured until the wound completely closes, and, for some studies, this has not occurred until many months after maggot therapy was discontinued. The full clinical benefits of maggot therapy may be best realized when treatments are continued as a method of maintenance debridement, that is, beyond the point of simple debridement.

Four- and six-arm clinical studies are proposed to test this hypothesis. As a multicenter study, it should be possible to assemble, quickly, the large number of subjects needed to attain the necessary power. If desired, the study could also assess the clinical advantages of free range maggot dressings over containment dressings, the latter of which provides wounds with maggot-derived chemicals but not the physical contact (“plowing action”) nor the capacity to benefit from the maggots' propensity to congregate in crevices, sinus tracts, and any other areas of greatest need.

It may be asked: why use maggot therapy as a maintenance debridement modality instead of other current methods? This question can, and should, also be answered by the proposed clinical study. Although a detailed discussion of study methods, eligibility criteria, choice of control modalities, measurable endpoints, and so forth, is beyond the scope of this treatise, one can certainly test the hypothesis that maggot therapy is not only more effective and efficient than other currently used debridement methods but also safer and less destructive to healthy tissue that would be growing during the proliferative phase of wound healing, while maintenance debridement is underway. Cost effectiveness, disinfection (the effect on microbial flora and clinical infection over time), and the effect of maggot therapy on short- and long-term quality of life could and should also be part of such a study.

By carefully planning our future clinical studies—pooling multi-institutional resources if necessary—we can maximize the impact and clinical relevance of these studies, while minimizing their overall expense. Until such studies are performed, clinicians can continue to use maggot therapy with confidence, at least for wound debridement and maintenance debridement. We are now also confident in the maggot's capacity to push the infected or simply stagnant “clean” wound to and through cellular proliferation and healing.

## Figures and Tables

**Table 1:** Publications identified and retrieved for review.

Study design	Number of publications identified	Number of publications retrieved and reviewed
Randomized clinical trial (RCT)	3	3
Nonrandomized, prospectively collected data, with control group	4	4
Nonrandomized, prospectively collected data, without control group	1	1
Controlled retrospectively collected data	1	1
Case series; no controls	20	18
Basic science	68	66

Total	97	93

**Table 2:** Wound Healing results associated with selected published maggot therapy studies.

	Pressure ulcer study^1^		Diabetic ulcer study^2^	
	Conventional therapy	MDT	Conventional therapy	MDT
Quality of wound base				
Initial granulation tissue as % of total area	31%	27%	18	19
Granulation tissue at 4 weeks^∗+^	29%	69%	15	56
Percentage of wounds developing ≥ 50% granulation tissue	18	51		
Weeks until granulation tissue reached > 50%	4.7	2.1		
Change in % of granulation tissue per week*	3.30%	13%		
Wound size and healing				
Initial surface area in sq cm*	14	22.1	6.3	13.3
Change in surface area during treatment (sq cm)^∗+^	6.3	−7.3	5	−3.8
Change in surface area per weeks^∗+^	1.4	−1.5	1.15	−0.78
Percentage of wounds which decreased in size within 4 weeks*	44%	79%		
Healing rate at 4 weeks^∗+^	−0.038	0.101	−0.08	0.08
Healing rate at 8 weeks^∗+^	−0.027	0.096	−0.02	0.07
Percentage of wounds completely healed	21%	39%	21	36
Average time to complete healing (weeks)	13.4	12	18	15

^1^Sherman, 2002 [18] (**∗**identifies significantly different results between the two arms of this study); ^2^Sherman, 2003 [19] (^+^identifies significantly different results between the two arms of this study). The wound healing rate, based on studies by Gilman [69] and Margolis et al. [70], was defined as the change in surface area divided by the mean circumference over time. Study details provided in text.
